# Efficacy of Low‐Dose Fluconazole for Primary Prophylaxis of Invasive Candida Infections in Patients With Acute Leukemia: A Double‐Blind Randomized Clinical Trial

**DOI:** 10.1002/cam4.70837

**Published:** 2025-03-28

**Authors:** Roghayeh Savary‐Kouzehkonan, Kourosh Sadeghi, Soroush Rad, Neda Alijani, Zohreh Baseri, Mohammad Vaezi, Seyed Asadollah Mousavi, Bita Shahrami

**Affiliations:** ^1^ Department of Clinical Pharmacy, School of Pharmacy Tehran University of Medical Sciences Tehran Iran; ^2^ Hematology, Oncology, and Stem Cell Transplantation Research Center, Research Institute for Oncology, Hematology, and Cell Therapy Tehran University of Medical Sciences Tehran Iran; ^3^ Department of Infectious Disease, School of Medicine Tehran University of Medical Sciences Tehran Iran; ^4^ Department of Microbiology, School of Medicine Iran University of Medical Sciences Tehran Iran; ^5^ Research Center for Rational Use of Drugs Tehran University of Medical Sciences Tehran Iran

**Keywords:** acute leukemia, antifungal prophylaxis, Candida, fluconazole, hematological malignancy, invasive fungal infection

## Abstract

**Background:**

Invasive fungal infections (IFIs), particularly Candida infections, are a significant cause of morbidity and mortality in patients with acute leukemia. While fluconazole is widely used for prophylaxis, the optimal dosing regimen remains uncertain. This study aimed to evaluate the efficacy of low‐dose fluconazole for primary prophylaxis against invasive Candida infections in patients with acute leukemia receiving intensive chemotherapy.

**Methods:**

A double‐blind, randomized clinical trial was conducted with patients diagnosed with acute leukemia. Patients were assigned to receive either low‐dose (150 mg/day) or standard high‐dose (400 mg/day) fluconazole for primary prophylaxis against invasive Candida infections during intensive chemotherapy. The primary outcomes were the efficacy of antifungal prophylaxis and the safety profile.

**Results:**

A total of 120 patients (60 per group) were enrolled. The overall incidence of Candida infections was similar between the groups (*p* = 0.615). Candida colonization was higher in the low‐dose fluconazole group during the first week, particularly with non‐albicans Candida at oral and subaxillary sites (*p* < 0.001). However, by the third week, both groups showed a significant decline in colonization, with the reduction in the oral cavity being statistically significant (*p* = 0.03). Aspergillosis occurred in 38.3% of patients, with no significant difference between groups (*p* > 0.99). Adverse events were similar in both groups (*p* > 0.05).

**Conclusion:**

Low‐dose fluconazole is an effective alternative to high‐dose regimens for preventing Candida infections in acute leukemia patients, with similar efficacy and safety. The rising threat of aspergillosis highlights the need for targeted prophylaxis. Further research is needed to refine strategies for high‐risk patients.

**Trial Registration:**

Iranian Registry of Clinical Trials (IRCT) number: IRCT20140818018842N37

## 1 Introduction

Patients with acute leukemia undergoing intensive chemotherapy are at a high risk of invasive fungal infections (IFIs), which are associated with significant morbidity and mortality [1, 2]. The immunosuppressed state resulting from severe and prolonged neutropenia, frequent use of broad‐spectrum antibiotics, and repeated chemotherapy cycles compounds this vulnerability [3]. Among IFIs, invasive Candida infections are a major concern, particularly in patients with hematological malignancies [4, 5]. *Candida* species such as *C. glabrata* and *C. parapsilosis* are increasingly reported as the predominant pathogens in regions such as Europe and the United States, with a clinical response rate of only 80%–85% to antifungal therapies in immunocompromised patients [6, 7, 8].

Antifungal prophylaxis has become a cornerstone of infection management in at‐risk patients [9]. Fluconazole, endorsed by the Infectious Diseases Society of America as the first‐line agent for prophylaxis and treatment of candidiasis, is widely favored due to its safety profile, oral bioavailability, affordability, and efficacy. Its mechanism of action, involving inhibition of ergosterol synthesis, disrupts fungal cell membrane integrity, making it effective against Candida infections [10, 11]. However, the optimal dosing regimen for fluconazole prophylaxis remains uncertain, especially in the context of invasive infections [12]. High‐dose regimens (400 mg daily) are commonly used to prevent invasive candidiasis in neutropenic patients, while lower doses (e.g., 100 mg) are often reserved for superficial infections [12, 13]. Despite this, recent evidence suggests that lower doses might still offer protection against invasive Candida infections, challenging the need for high‐dose regimens in all cases.

Geographical differences in the incidence and etiology of IFIs, coupled with variability in antifungal prophylaxis practices and patient demographics, further complicate the establishment of standardized guidelines [14]. As a result, clinicians face uncertainty regarding the most effective and safe prophylactic strategies, particularly for patients with acute leukemia undergoing intensive chemotherapy.

Given these challenges, this study aimed to evaluate the efficacy of low‐dose fluconazole for primary prophylaxis against invasive Candida infections in patients with acute leukemia receiving intensive chemotherapy. By addressing this question, the study seeks to inform clinical practice and potentially identify a more cost‐effective and safer prophylactic approach for this high‐risk population.

## 2 Methods

### 2.1 Study Design and Setting

This study was a single‐center, randomized, double‐blind clinical trial conducted at the Research Institute for Oncology, Hematology, and Cell Therapy, affiliated with Tehran University of Medical Sciences (TUMS), in Iran, from January 2023 to August 2023.

### 2.2 Study Population

This study included adult patients with acute leukemia receiving intensive chemotherapy who were candidates for primary prophylaxis against Candida. Patients were excluded from the study based on the following criteria: a history of idiosyncratic allergic reactions to azoles; receipt of systemic antifungal treatment within the previous 2 weeks; a confirmed fungal infection; pregnancy or breastfeeding; the use of medications with significant clinical interactions (category X) with fluconazole; a history of IFIs requiring systemic treatment within the last 6 months; or an estimated life expectancy of less than 3 weeks. Additionally, patients were excluded if they developed a severe hypersensitivity reaction to azoles; had liver dysfunction (defined as transaminases level > 10 times the upper limit of normal) [15]; renal failure (creatinine clearance of ≤ 50 mL/min); QTc interval prolongation or Torsades de Pointes [16]; or non‐adherence to fluconazole for more than three consecutive days during the study period [17].

### 2.3 Randomization and Blinding

In this study, randomization was conducted using the Clinical Trial Randomization Tool provided by the National Cancer Institute (NCI) (available at https://ctrandomization.cancer.gov/tool/). A total of 120 participants were randomly assigned to one of two equal groups (60 patients per group) using a web‐based algorithm. The study was double‐blind, ensuring that both investigators and participants were unaware of group assignments. The randomization results were securely placed in sealed envelopes, which were sequentially numbered. Each participant was assigned a fluconazole dose based on the envelope drawn at the time of enrollment, ensuring allocation concealment.

### 2.4 Study Protocol

Participants in Group 1 received 400 mg of fluconazole once daily, while those in Group 2 received a lower dose of 150 mg once daily, both administered orally, from the initiation of intensive chemotherapy until the resolution of neutropenia or prophylaxis failure. Daily monitoring included assessing medication adherence, recording adverse effects, and evaluating potential drug interactions. Kidney and liver function tests were performed at least three times per week. Fungal colonization was monitored through weekly cultures for Candida, using oropharyngeal, femoral, and subaxillary swabs, which were cultured on Sabouraud dextrose agar. If a fungal infection was suspected—such as in febrile neutropenic patients unresponsive to antibiotics—an evaluation for fungal infection was promptly conducted. This evaluation included fungal cultures (from blood, urine, and central line samples), galactomannan testing, and imaging of suspected sites of infection. The study continued until prophylaxis was completed or failure occurred.

### 2.5 Definition of Antifungal Prophylaxis and Failure

Primary antifungal prophylaxis was defined as the preventive administration of antifungal agents to reduce the risk of IFIs in patients with acute leukemia who were expected to experience severe and potentially fatal neutropenia, defined as an absolute neutrophil count (ANC) of less than 500/μL for more than 7 days [18], placing them at high risk for fungal infections. Prophylaxis failure was indicated by at least one of the following: a confirmed diagnosis of an IFI, or the need for systemic antifungal therapy for more than 4 days due to a suspected probable or possible fungal infection.

### 2.6 Classification of Fungal Infections Indicating Antifungal Prophylaxis Failure

During the prophylaxis period, all patients were closely monitored for the development of any fungal infections based on clinical evaluation and fungal colonization and culture. Infections were classified as follows:

*Candida colonization*: *Candida* species were isolated from three distinct non‐blood body sites: the oral cavity, subaxillary region, and femoral area—over a period of 3 weeks.

*Superficial candidiasis*: This category includes Candida infections of the skin, mouth, pharynx, or genital tract, identified by positive cultures but without systemic symptoms. These infections typically present as localized lesions or oral thrush [19].

*Systemic fungal infection*: Diagnosis when significant clinical evidence of fungal infection is found in tissue or blood, confirmed by culture or biopsy from the affected site, and supported by imaging findings (e.g., lungs, sinuses, or other tissues). Elevated biochemical markers, such as galactomannan levels, further support this diagnosis [20, 21].

*Probable fungal infection favoring Candida*: Defined by at least one of the following: (1) fever of unknown origin unresponsive to broad‐spectrum antibiotics, without evidence of other fungal infections, or (2) a Candida score of two or higher, necessitating empirical antifungal treatment for presumed Candida infection [22, 23].

*Possible Aspergillus infection*: Applied to immunocompromised patients or those on immunosuppressive therapy who exhibit characteristic CT scan findings of Aspergillus infection in the lungs, sinuses, or other organs. If these findings are accompanied by a positive Aspergillus culture from non‐sterile specimens (e.g., bronchoalveolar lavage or saliva) or a positive galactomannan test, the diagnosis is upgraded to probable Aspergillus infection [24, 25]. These diagnostic criteria were established in consultation with infectious disease specialists.

### 2.7 Safety Monitoring and Management

The onset, duration, severity, and potential relationship of any side effects to the administered fluconazole were meticulously documented. In response to the patient's clinical condition, the drug dosage could be adjusted over a period of up to 3 days. Temporary discontinuation of the drug was mandated under the following circumstances: an increase in liver enzymes exceeding three times the upper limit of normal, accompanied by symptoms of liver damage; an increase in liver enzymes exceeding five times the upper limit of normal [26]; and QTc interval prolongation exceeding 500 ms [16]. Once these conditions were resolved, treatment could be resumed after a maximum of 3 days. If the conditions did not improve, the patient was excluded from the study.

### 2.8 Statistical Analysis

Data collected by the researcher using the questionnaire tool were systematically recorded and organized with Excel software. Subsequently, the data were analyzed using SPSS software (version 26), employing both descriptive and inferential statistical methods. Continuous variables were assessed using either Student's t‐test or the Wilcoxon signed‐rank test, depending on the distribution of the data. For categorical variables, chi‐square tests were utilized. Statistical significance was determined at a 95% confidence level, with p‐values less than 0.05 considered statistically significant.

## 3 Results

### 3.1 Patients' Characteristics

**FIGURE 1:**
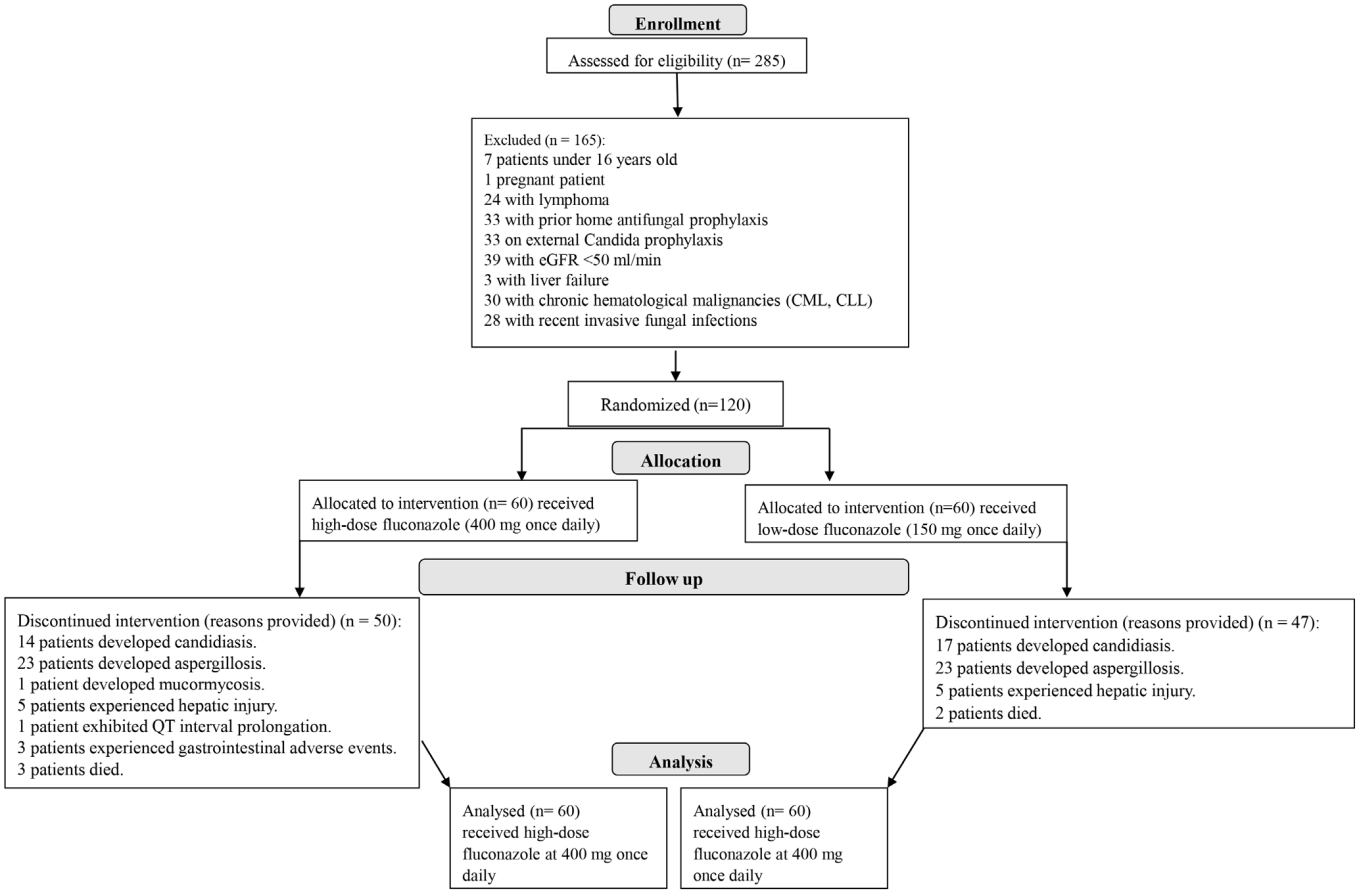
Flowchart of inclusion and exclusion criteria.

Throughout the study period, a total of 285 patients were screened, leading to the inclusion of 120 patients (60 in Group 1 and 60 in Group 2), as depicted in Figure 1.

Among the enrolled patients, 33 (27.5%) had acute lymphoblastic leukemia (ALL), while 87 (72.5%) were diagnosed with acute myeloblastic leukemia (AML). The majority of patients were male, comprising 76 (63.3%). Demographic information is detailed in Table 1.

**TABLE 1:** Baseline characteristics of study patients (*N* = 120).

Parameter	Group 1; fluconazole 400 mg/day (*N* = 60)	Group 2; fluconazole 150 mg/day (*N* = 60)	*p*
Age (years); mean (range)	40.2 (18–63)	40.8 (18–71)	0.803
Height (cm); mean ± SD	168.1 ± 12.3	171.2 ± 8.9	0.114
Weight (kg); mean ± SD	75.4 ± 20.1	72.3 ± 13.5	0.383
Type of hematological malignancy; *N* (%)			
ALL	13 (21.7)	20 (33.3)	0.152
AML	47 (78.3)	40 (66.7)	
Comorbidities; *N* (%)			
Heart failure	2 (3.3%)	2 (3.3%)	0.055
CKD	0	3 (5.0%)	
Diabetes mellitus	2 (3.3%)	3 (5.0%)	
Hypertension	4 (6.7%)	6 (10.0%)	
Hypothyroidism	8 (13.3%)	2 (3.3%)	
Dyslipidemia	2 (3.3%)	0	
Hepatitis B	2 (3.3%)	0	
ACS	0	3 (5.0%)	
Other cancer	2 (3.3%)	3 (5.0%)	
Seizure	0	1 (1.7%)	
Days of prophylaxis; mean (range)	18.3 (3–48)	19.5 (3–0)	0.407
Discharge; *N* (%)	56 (93.3)	54 (90.0)	0.509
Death; *N* (%)	4 (6.7)	6 (10.0)	

Abbreviations: ACS, acute coronary syndromes; ALL, acute lymphocyte leukemia; AML, acute myeloid leukemia; CKD, chronic kidney disease; SD, standard deviation.

Of the 120 patients enrolled, 110 were successfully discharged from the hospital, while 10 patients regrettably passed away during their hospitalization.

### 3.2 Efficacy of Antifungal Prophylaxis

**FIGURE 2:**
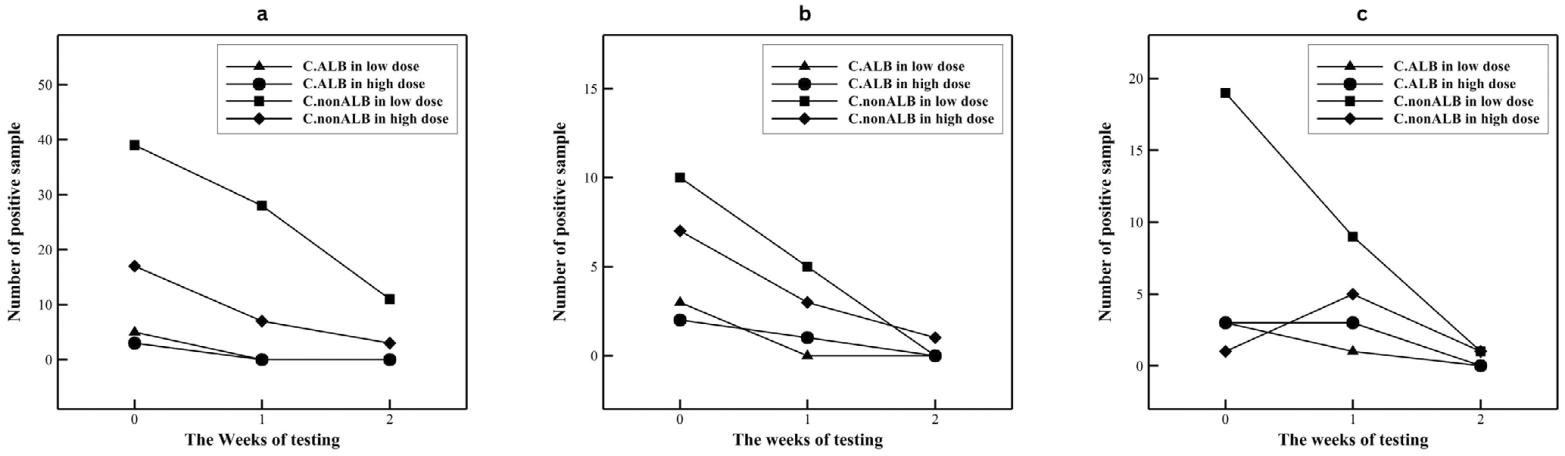
Colonization trends of *Candida albicans* and non‐*albicans Candida* in three consecutive weekly samplings: (a) oral cavity, (b) subaxillary region, and (c) femoral area in two treatment groups.

In the first week, *Candida albicans* colonization was higher in Group 2, while non‐*albicans Candida* colonization was significantly higher in Group 2 at the oral and subaxillary sites (*p* < 0.001). Across the following weeks, both groups showed a decline in colonization, with statistically significant changes observed in the third week at the oral cavity (*p* = 0.03). The trends are shown in Figure 2, with subfigures a, b, and c representing the oral cavity, subaxillary, and femoral regions, respectively.

A total of 31 patients (25.8%) developed Candida infections as a failure of primary prophylaxis, with 14 patients (23.3%) in Group 1 and 17 patients (28.3%) in Group 2, showing no significant difference between the groups (*p* = 0.615). No cases of systemic candidiasis were diagnosed in either group receiving fluconazole prophylaxis. Aspergillosis was observed in 46 patients (38.3%), with 23 patients (38.3%) in each group. Additionally, 1 patient in Group 2 was diagnosed with mucormycosis. Detailed outcomes are provided in Table 2. In the subgroup analysis, no significant correlation was found between the two types of acute leukemia, ALL and AML, for these outcomes (Table 3).

**TABLE 2:** Fungal infections as indicators of prophylaxis failure in study patients (*N* = 120).

Outcome measure	Group 1; fluconazole 400 mg/day (*N* = 60)	Group 2; fluconazole 150 mg/day (*N* = 60)	*p*
Candida Infections			
Candidemia	0 (0)	0 (0)	0
Probable candidiasis	13 (21.7)	15 (25.0)	0.666
Superficial candidiasis	1 (1.7)	2 (3.3)	> 0.99
Aspergillus infections			
Proven aspergillosis	2 (3.3)	1 (1.7)	> 0.99
Probable aspergillosis	16 (26.7)	18 (30.0)	0.685
Possible aspergillosis	5 (8.3)	4 (6.7)	> 0.99
Other fungal infections			
Mucormycosis	0 (0)	1 (1.7)	> 0.99

**TABLE 3:** Fungal infections in patients receiving fluconazole prophylaxis by hematologic malignancy (ALL vs. AML).

Fungal infection	Hematologic malignancy	Group 1; fluconazole 400 mg/day (*N* = 60)	Group 2; fluconazole 150 mg/day (*N* = 60)	*p*
Candidemia	ALL	0 (0%)	0 (0%)	0.183
	AML	0 (0%)	0 (0%)	
Superficial candidiasis	ALL	1 (7.7%)	1 (5%)	0.183
	AML	0 (0%)	1 (2.5%)	
Probable candidiasis	ALL	1 (7.7%)	8 (40%)	0.053
	AML	12 (25.5%)	7 (17.5%)	
Proven aspergillosis	ALL	0 (0%)	0 (0%)	0.560
	AML	2 (4.3%)	1 (2.5%)	
Probable aspergillosis	ALL	3 (23.1%)	5 (25.0%)	0.540
	AML	13 (27.7%)	13 (32.5%)	
Possible aspergillosis	ALL	1 (7.7%)	2 (10%)	0.705
	AML	3 (6.4%)	3 (7.5%)	
Mucormycosis	ALL	0 (0%)	1 (1.1%)	> 0.99
	AML	0 (0%)	0 (0%)	

Abbreviations: ALL, acute lymphoblastic leukemia; AML, acute myeloblastic leukemia.

### 3.3 Safety

Gastrointestinal effects, hepatic impairment, and QT prolongation were reported as significant drug‐related effects in all patients; however, none of these adverse events showed significant differences between the two groups (all *p* > 0.05). Adverse events led to the withdrawal of fluconazole in 8 patients (6.6%): 5 patients (8.3%) from Group 1 and 3 patients (5%) from Group 2. However, the difference between the groups was not statistically significant (*p* = 0.113). During the prophylactic regimen, 5 patients (4.1%) died: 3 patients (5%) from Group 1 and 2 patients (3.3%) from Group 2 (*p* > 0.99). Details of the significant adverse effects associated with fluconazole prophylaxis are summarized in Table 4.

**TABLE 4:** Significant adverse reactions to fluconazole prophylaxis in study patients (*N* = 120).

Safety concern		Group 1; fluconazole 400 mg/day (*N* = 60)	Group 2; fluconazole 150 mg/day (*N* = 60)	*p*
Gastrointestinal upset		3 (5.0)	0 (0)	0.244
Hepatic impairment	Transaminase > 3 × ULN with clinical symptoms	0 (0)	0 (0)	> 0.99
	Transaminase > 5 × ULN	1 (1.66)	2 (3.33)	
	Transaminase > 10 × ULN	4 (6.66)	3 (5.0)	
QT prolongation		1 (1.66)	0	> 0.99

Abbreviation: ULN, upper limit normal.

## 4 Discussion

The findings of this double‐blind, randomized clinical trial evaluating the efficacy of low‐dose fluconazole (150 mg daily) compared to the standard high‐dose regimen (400 mg daily) for primary prophylaxis of invasive Candida infections in patients with acute leukemia undergoing intensive chemotherapy provide valuable insights into antifungal prophylaxis in this high‐risk population. The study demonstrated no statistically significant differences between the two groups in terms of prophylaxis failure, prophylaxis discontinuation, systemic candidiasis, or overall safety profiles. These results suggest that a lower dose of fluconazole may provide comparable protection against invasive Candida infections while maintaining a similar safety profile, potentially offering a more cost‐effective and safer approach to antifungal prophylaxis.

The study observed that Candida colonization, particularly with non‐albicans Candida species, was initially higher in the low‐dose group but declined over time. By the third week of prophylaxis, colonization patterns were similar between the two groups. Importantly, systemic candidiasis was not observed in either group, demonstrating that both dosing regimens effectively prevented invasive Candida infections. These findings align with prior research, such as that by McMillan et al., which reported comparable efficacy of low‐dose (200 mg) and high‐dose (400 mg) fluconazole in reducing colonization and superficial and systemic Candida infections during the neutropenic phase in bone marrow transplant recipients [27]. Similar studies in hematopoietic stem cell transplantation (HSCT) settings have shown that fluconazole doses under 400 mg/day effectively suppress fungal colony formation and prevent invasive infections, comparable to higher doses, while also potentially reducing costs and minimizing side effects [28].

Our findings challenge traditional recommendations advocating high‐dose fluconazole for neutropenic patients, particularly given the rising prevalence of non‐albicans Candida species with variable susceptibility to fluconazole. Global guidelines often recommend a 400 mg/day dosage, but in Japan and other regions, lower doses (100–200 mg/day) are frequently used following HSCT, reflecting regional differences in practice [13, 29].

Safety monitoring revealed no significant differences in the incidence of adverse events such as gastrointestinal effects, hepatic impairment, or QT prolongation between the two groups. Drug discontinuation rates due to adverse effects and mortality were also comparable, reinforcing the safety of the low‐dose regimen. These findings support the use of low‐dose fluconazole, particularly for patients at risk of dose‐dependent toxicities [30].

Despite the comparable efficacy and safety profiles, several considerations warrant further exploration. Notably, the study did not show a reduction in the incidence of other IFIs, such as aspergillosis, which occurred at similar rates in both groups. This is particularly relevant given the widespread adoption of posaconazole prophylaxis in AML induction therapy in Western settings, where it has been shown to significantly reduce Aspergillus infections. However, in many regions, including those with limited access to newer antifungals due to economic or regulatory constraints, fluconazole remains the primary prophylactic option. Fluconazole remains a commonly used agent for antifungal prophylaxis in patients with acute leukemia, particularly in settings where access to broader‐spectrum azoles such as posaconazole is limited. While the NCCN guidelines recommend mold‐active azoles, particularly posaconazole, as the preferred prophylactic agent for AML patients [31], fluconazole is still widely used due to economic and regulatory barriers in many regions. Our study aimed to evaluate whether a lower dose of fluconazole could provide effective prophylaxis against invasive Candida infections, particularly in settings where posaconazole is not routinely available. Although our study included both AML and ALL patients, it is important to note that fluconazole remains a category 1 recommendation for prophylaxis in ALL. The inclusion of AML patients reflects real‐world clinical practice in regions where fluconazole is still the primary prophylactic agent despite guideline recommendations. Future studies may be needed to assess alternative prophylactic strategies, particularly in AML patients at high risk for invasive mold infections. One potential concern with low‐dose fluconazole prophylaxis is the risk of selecting for fluconazole‐resistant Candida strains, particularly among non‐albicans species. Prior studies have indicated that prolonged exposure to subtherapeutic fluconazole concentrations may contribute to the emergence of resistant strains [32]. While our study did not observe clinical fluconazole resistance, the transient increase in Candida colonization in the low‐dose group during the first week may suggest selective pressure favoring non‐albicans species with reduced fluconazole susceptibility. Given the increasing global incidence of fluconazole‐resistant Candida, particularly *Candida glabrata* and *Candida auris*, future research should evaluate the long‐term microbiological impact of low‐dose prophylaxis. Periodic antifungal susceptibility testing and resistance surveillance are essential to mitigate this risk. The findings underscore the importance of tailoring antifungal prophylaxis to the local epidemiology of fungal infections, as regional variations in the prevalence of *Aspergillus* and other molds may necessitate broader‐spectrum agents in certain settings. Moreover, the delay in initiating antifungal treatment significantly increases mortality, as even a 12‐ to 24‐h delay in antifungal intervention can double the crude mortality rate for candidemia, emphasizing the importance of timely and effective prophylaxis.

Additionally, no statistically significant correlation was found between the type of hematological malignancy and the type of fungal infection in the two groups, consistent with other studies showing no difference in the occurrence of IFIs between patients with ALL and AML [33]. However, some research has reported a higher prevalence of fungal infections among AML patients, suggesting that further subgroup analyses in larger cohorts are necessary [34, 35].

### 4.1 Study Limitations

This study was conducted at a single center, which may limit the generalizability of the findings to other populations or healthcare settings. The relatively small sample size may have affected the ability to detect less common fungal infections or subtle differences in clinical outcomes. Larger, multicenter trials are needed to validate these findings and explore their broader applications.

## 5 Conclusion

The results of this trial suggest that low‐dose fluconazole is a viable alternative to high‐dose regimens for primary prophylaxis of invasive Candida infections in acute leukemia patients undergoing intensive chemotherapy. With comparable efficacy and safety, the low‐dose approach offers the added benefits of cost‐effectiveness and a potentially lower risk of adverse effects. However, the study also highlights the growing threat of aspergillosis, suggesting the need for targeted prophylactic strategies for mold infections. Further research, particularly in diverse and larger populations, is necessary to confirm these findings and guide antifungal prophylaxis in high‐risk patients.

## Author Contributions

**Roghayeh Savary‐Kouzehkonan:** conceptualization (equal), data curation (lead), formal analysis (lead), investigation (lead), methodology (equal), project administration (equal), software (equal), visualization (lead), writing – original draft (lead). **Kourosh Sadeghi:** conceptualization (equal), data curation (supporting), formal analysis (supporting), investigation (supporting), methodology (supporting), project administration (equal), software (supporting), writing – review and editing (lead). **Soroush Rad:** conceptualization (equal), formal analysis (equal), investigation (supporting), methodology (supporting), writing – review and editing (supporting). **Neda Alijani:** data curation (equal), formal analysis (supporting), investigation (supporting), methodology (equal), writing – review and editing (equal). **Zohreh Baseri:** data curation (equal), formal analysis (supporting), methodology (supporting), writing – review and editing (equal). **Mohammad Vaezi:** conceptualization (equal), formal analysis (equal), investigation (equal), methodology (equal), project administration (equal), supervision (equal), writing – review and editing (equal). **Seyed Asadollah Mousavi:** conceptualization (equal), formal analysis (equal), investigation (equal), methodology (equal), supervision (equal), writing – review and editing (equal). **Bita Shahrami:** conceptualization (equal), data curation (supporting), formal analysis (equal), investigation (supporting), methodology (equal), project administration (equal), writing – review and editing (equal).

## Ethics Statement

The study protocol was approved by the TUMS ethics committee (IR.TUMS.TIPS.REC.1401.040).

## Consent

Informed consent was obtained from all participants.

## Conflicts of Interest

The authors declare no conflicts of interest.

## Data Availability

Data are available from the authors upon reasonable request.

## References

[1] Y. Sun , J. Hu , H. Huang , et al., “Fluconazole Is as Effective as Other Anti‐Mold Agents in Preventing Early Invasive Fungal Disease After Allogeneic Stem Cell Transplantation: Assessment of Antifungal Therapy in Haematological Disease in China,” Translational Cancer Research 9, no. 11 (2020): 6900–6911. 35117298 10.21037/tcr-19-2887 PMC8798361

[2] J. Xia , Z. Wang , T. Li , F. Lu , D. Sheng , and W. Huang , “Immunosuppressed Patients With Clinically Diagnosed Invasive Fungal Infections: The Fungal Species Distribution, Antifungal Sensitivity and Associated Risk Factors in a Tertiary Hospital of Anhui Province,” Infection and Drug Resistance 15 (2022): 321–333. 35140478 10.2147/IDR.S351260 PMC8818762

[3] F. Moreno‐Sanchez and B. Gomez‐Gomez , “Antibiotic Management of Patients With Hematologic Malignancies: From Prophylaxis to Unusual Infections,” Current Oncology Reports 24, no. 7 (2022): 835–842. 35316843 10.1007/s11912-022-01226-y PMC8938218

[4] X. Gong , M. Yang , D. Lin , et al., “Candidemia in Patients With Acute Leukemia: Analysis of 7 Years' Experience at a Single Center in China,” Mediterranean Journal of Hematology and Infectious Diseases 12, no. 1 (2020): e2020003. 31934313 10.4084/MJHID.2020.003 PMC6951356

[5] J. Signorelli , M. Lei , J. Lam , et al., “Incidence of Invasive Fungal Infections in Acute Myeloid Leukemia Without Antifungal Prophylaxis,” Clinical Lymphoma Myeloma and Leukemia 20, no. 11 (2020): e883–e889, 10.1016/j.clml.2020.06.008. 32917574

[6] M. N. Gamaletsou , T. J. Walsh , T. Zaoutis , et al., “A Prospective, Cohort, Multicentre Study of Candidaemia in Hospitalized Adult Patients With Haematological Malignancies,” Clinical Microbiology and Infection 20, no. 1 (2014): O50–O57, 10.1111/1469-0691.12312. 23889746

[7] C. Keighley , L. Cooley , A. J. Morris , et al., “Consensus Guidelines for the Diagnosis and Management of Invasive Candidiasis in Haematology, Oncology and Intensive Care Settings, 2021,” Internal Medicine Journal 51, no. S7 (2021): 89–117. 34937142 10.1111/imj.15589

[8] N. V. Sipsas , R. E. Lewis , J. Tarrand , et al., “Candidemia in Patients With Hematologic Malignancies in the Era of New Antifungal Agents (2001–2007): Stable Incidence but Changing Epidemiology of a Still Frequently Lethal Infection,” Cancer 115, no. 20 (2009): 4745–4752. 19634156 10.1002/cncr.24507

[9] R. Sprute , J. A. Nacov , D. Neofytos , et al., “Antifungal Prophylaxis and Pre‐Emptive Therapy: When and How?,” Molecular Aspects of Medicine 92 (2023): 101190. 37207579 10.1016/j.mam.2023.101190

[10] P. G. Pappas , C. A. Kauffman , D. R. Andes , et al., “Clinical Practice Guideline for the Management of Candidiasis: 2016 Update by the Infectious Diseases Society of America,” Clinical Infectious Diseases 62, no. 4 (2015): e1–e50. 26679628 10.1093/cid/civ933 PMC4725385

[11] S. A. Sayed , E. A. B. Hassan , M. R. Abdel Hameed , et al., “Ketorolac‐Fluconazole: A New Combination Reverting Resistance in *Candida albicans* From Acute Myeloid Leukemia Patients on Induction Chemotherapy: In Vitro Study,” Journal of Blood Medicine 12 (2021): 465–474, 10.2147/JBM.S302158. 34163275 PMC8214543

[12] D. Vuichard , M. Weisser , C. Orasch , et al., “Weekly Use of Fluconazole as Prophylaxis in Haematological Patients at Risk for Invasive Candidiasis,” BMC Infectious Diseases 14 (2014): 573, 10.1186/s12879-014-0573-5. 25384689 PMC4233028

[13] J. L. Goodman , D. J. Winston , R. A. Greenfield , et al., “A Controlled Trial of Fluconazole to Prevent Fungal Infections in Patients Undergoing Bone Marrow Transplantation,” New England Journal of Medicine 326, no. 13 (1992): 845–851. 1542320 10.1056/NEJM199203263261301

[14] M. D. Bergamasco , C. A. P. Pereira , C. Arrais‐Rodrigues , et al., “Epidemiology of Invasive Fungal Diseases in Patients With Hematologic Malignancies and Hematopoietic Cell Transplantation Recipients Managed With an Antifungal Diagnostic Driven Approach,” Journal of Fungi 7, no. 8 (2021): 588. 34436127 10.3390/jof7080588 PMC8397156

[15] C. Van Delden , D. P. Lew , B. Chapuis , P. Rohner , and B. Hirschel , “Antifungal Prophylaxis in Severely Neutropenic Patients: How Much Fluconazole Is Necessary?,” Clinical Microbiology and Infection 1, no. 1 (1995): 24–30. 11866717 10.1111/j.1469-0691.1995.tb00020.x

[16] M. Salem , T. Reichlin , D. Fasel , and A. Leuppi‐Taegtmeyer , “Torsade de Pointes and Systemic Azole Antifungal Agents: Analysis of Global Spontaneous Safety Reports,” Global Cardiology Science & Practice 2017, no. 2 (2017): 11. 29644223 10.21542/gcsp.2017.11 PMC5871400

[17] P. G. Pappas , C. A. Kauffman , D. Andes , et al., “Clinical Practice Guidelines for the Management of Candidiasis: 2009 Update by the Infectious Diseases Society of America,” Clinical Infectious Diseases 48, no. 5 (2009): 503–535, 10.1086/596757. 19191635 PMC7294538

[18] B. J. Kullberg and M. C. Arendrup , “Invasive Candidiasis,” New England Journal of Medicine 373, no. 15 (2015): 1445–1456. 26444731 10.1056/NEJMra1315399

[19] P. S. Dabas , “An Approach to Etiology, Diagnosis and Management of Different Types of Candidiasis,” Journal of Yeast and Fungal Research 4, no. 6 (2013): 63–74.

[20] N. Barantsevich and E. Barantsevich , “Diagnosis and Treatment of Invasive Candidiasis,” Antibiotics 11, no. 6 (2022): 718, 10.3390/antibiotics11060718. 35740125 PMC9219674

[21] J. R. Perfect , “Fungal Diagnosis: How Do We Do It and Can We Do Better?,” Current Medical Research and Opinion 29, no. S4 (2013): 3–11. 10.1185/03007995.2012.761134 23621588

[22] P. Eggimann , Y.‐A. Que , J.‐P. Revelly , and J.‐L. Pagani , “Preventing Invasive Candida Infections. Where Could We Do Better?,” Journal of Hospital Infection 89, no. 4 (2015): 302–308. 25592726 10.1016/j.jhin.2014.11.006

[23] B. Posteraro , G. De Pascale , M. Tumbarello , et al., “Early Diagnosis of Candidemia in Intensive Care Unit Patients With Sepsis: A Prospective Comparison of (1→3)‐β‐D‐Glucan Assay, Candida Score, and Colonization Index,” Critical Care 15, no. 5 (2011): 1–10, 10.1186/cc10507. PMC3334800 22018278

[24] F. Lamoth and T. Calandra , “Pulmonary Aspergillosis: Diagnosis and Treatment,” European Respiratory Review 31, no. 166 (2022): 220114. 36450372 10.1183/16000617.0114-2022 PMC9724826

[25] S. Quereshi , P. Paralikar , R. Pandit , M. Razzaghi‐Abyaneh , K. Kon , and M. Rai , “Pulmonary Aspergillosis: Diagnosis and Treatment,” in The Microbiology of Respiratory System Infections (Elsevier, 2016), 167–183.

[26] A. Rakhshan , B. R. Kamel , A. Saffaei , and M. Tavakoli‐Ardakani , “Hepatotoxicity Induced by Azole Antifungal Agents: A Review Study,” Iranian Journal of Pharmaceutical Research 22, no. 1 (2023): e130336, 10.5812/ijpr-130336. 38116543 PMC10728840

[27] M. L. MacMillan , J. L. Goodman , T. E. DeFor , and D. J. Weisdorf , “Fluconazole to Prevent Yeast Infections in Bone Marrow Transplantation Patients: A Randomized Trial of High Versus Reduced Dose, and Determination of the Value of Maintenance Therapy,” American Journal of Medicine 112, no. 5 (2002): 369–379. 11904111 10.1016/s0002-9343(01)01127-5

[28] K. Hirade , S. Kusumoto , H. Hashimoto , et al., “Low‐Dose Fluconazole as a Useful and Safe Prophylactic Option in Patients Receiving Allogeneic Hematopoietic Stem Cell Transplantation,” Cancer Medicine 13, no. 3 (2024): e6815. 38213090 10.1002/cam4.6815 PMC10905229

[29] O. Imataki , M. Kami , S. W. Kim , et al., “A Nationwide Survey of Deep Fungal Infections and Fungal Prophylaxis After Hematopoietic Stem Cell Transplantation in Japan,” Bone Marrow Transplantation 33, no. 12 (2004): 1173–1179. 15094754 10.1038/sj.bmt.1704526

[30] R. Bellmann and P. Smuszkiewicz , “Pharmacokinetics of Antifungal Drugs: Practical Implications for Optimized Treatment of Patients,” Infection 45, no. 6 (2017): 737–779. 28702763 10.1007/s15010-017-1042-z PMC5696449

[31] L. R. Baden , S. Swaminathan , M. Angarone , et al., “Prevention and Treatment of Cancer‐Related Infections, Version 2.2016, NCCN Clinical Practice Guidelines in Oncology,” Journal of the National Comprehensive Cancer Network 14, no. 7 (2016): 882–913, 10.6004/jnccn.2016.0093. 27407129

[32] L. P. Brion , S. E. Uko , and D. L. Goldman , “Risk of Resistance Associated With Fluconazole Prophylaxis: Systematic Review,” Journal of Infection 54, no. 6 (2007): 521–529. 17239952 10.1016/j.jinf.2006.11.017

[33] M. Sezgin Evim , Ö. Tüfekçi , B. Baytan , et al., “Invasive Fungal Infections in Children With Leukemia: Clinical Features and Prognosis,” Turkish Journal of Hematology 39, no. 2 (2022): 94–102. 34792308 10.4274/tjh.galenos.2021.2021.0203 PMC9160694

[34] Z. Kaya , T. Gursel , U. Kocak , Y. Z. Aral , A. Kalkanci , and M. Albayrak , “Invasive Fungal Infections in Pediatric Leukemia Patients Receiving Fluconazole Prophylaxis,” Pediatric Blood & Cancer 52, no. 4 (2009): 470–475. 19058205 10.1002/pbc.21868

[35] S.‐M. Oh , J. M. Byun , E. Chang , et al., “Incidence of Invasive Fungal Infection in Acute Lymphoblastic and Acute Myelogenous Leukemia in the Era of Antimold Prophylaxis,” Scientific Reports 11, no. 1 (2021): 22160. 34773060 10.1038/s41598-021-01716-2 PMC8590008

